# Contemporary Trends in Candidemia: Mortality, Species, and Antifungal Resistance Across Three Periods (2017–2025) in Houston, Texas

**DOI:** 10.1093/ofid/ofag558

**Published:** 2026-09-09

**Authors:** Mary North Jones, Masayuki Nigo, María Alejandra Pérez, Stefano Casarin, Aarjav Sanghvi, Stephen L Jones, David B Corry, Cesar A Arias, Max W Adelman

**Affiliations:** Division of Infectious Diseases, Department of Medicine, Houston Methodist Hospital, Houston, Texas, USA; Department of Medicine, Baylor College of Medicine, Houston, Texas, USA; Division of Infectious Diseases, Department of Medicine, Houston Methodist Hospital, Houston, Texas, USA; Center for Infectious Diseases, Houston Methodist Research Institute, Houston, Texas, USA; Department of Medicine, Weill Cornell Medical College, New York, New York, USA; Center for Infectious Diseases, Houston Methodist Research Institute, Houston, Texas, USA; Center for Precision Surgery, Houston Methodist Research Institute, Houston, Texas, USA; LaSIE, UMR 7356, CNRS, La Rochelle Université, La Rochelle, France; Department of Surgery, Weill Cornell Medical College, New York, New York, USA; Center for Health Data Science and Analytics, Houston Methodist Hospital, Houston, Texas, USA; Center for Health Data Science and Analytics, Houston Methodist Hospital, Houston, Texas, USA; Department of Medicine, Baylor College of Medicine, Houston, Texas, USA; Department of Pathology and Immunology, Baylor College of Medicine, Houston, Texas, USA; Michael E. DeBakey Department of Veterans Affairs Medical Center, Center for Translational Research on Inflammatory Diseases (CTRID), Houston, Texas, USA; Division of Infectious Diseases, Department of Medicine, Houston Methodist Hospital, Houston, Texas, USA; Center for Infectious Diseases, Houston Methodist Research Institute, Houston, Texas, USA; Department of Medicine, Weill Cornell Medical College, New York, New York, USA; Division of Infectious Diseases, Department of Medicine, Houston Methodist Hospital, Houston, Texas, USA; Center for Infectious Diseases, Houston Methodist Research Institute, Houston, Texas, USA; Department of Medicine, Weill Cornell Medical College, New York, New York, USA; Division of Pulmonary, Critical Care, and Sleep Medicine, Houston Methodist Hospital, Houston, Texas, USA

**Keywords:** Antifungal resistance, *C. auris*, Candidemia, Fungal infection

## Abstract

**Background:**

*Candida* species distribution has changed and antifungal resistance has increased since COVID-19. We determined whether these trends contributed to changes in candidemia mortality.

**Methods:**

In a retrospective cohort study of patients with candidemia in Houston, TX, we compared clinical characteristics, species, and resistance between 3 periods (P1, 2017–2019; P2, 2020–2022; and P3, 2023–2025) and assessed contribution to mortality. We compared 30-day mortality in candidemia versus bacteremia.

**Results:**

Among 1275 patients with candidemia (1386 episodes), 30-day mortality increased from P1 to P2 (19.7% vs 31.9%, *P* < .001) and remained above baseline in P3 (29.8%, *P* < .001 vs P1). In contrast, 30-day mortality increased from P1 to P2 in patients with Enterobacterales (7.4% vs 9.5%, *P* = .004) and *Staphylococcus aureus* bacteremia (9.5% vs 14.3%, *P* < .001) but returned to baseline in P3 (both *P* > .05 vs P1). Mean age (62.7 ± 16.0 vs 66.1 ± 14.8, *P* = .002) and median Elixhauser Comorbidity Index (8 [IQR 5–12] vs 11 [IQR 6–15], *P* < .001) increased from P1 to P3 among candidemia patients. Proportion of ICU-onset candidemia increased from P1 to P2 (31.3% vs 40.6%, *P* = .02). Proportion of *C. auris* increased from P1 to P3 (0.5% vs 20.5%, *P* < .001). In multivariable analyses, age (OR 1.03, 95% CI 1.02–1.04) and ICU-onset candidemia (OR 2.08, 95%CI 1.46–2.98) were associated with increased mortality. In univariable analyses, *C. auris* and antifungal resistance were not associated with increased mortality.

**Conclusions:**

Increased post-candidemia mortality was associated in part with demographic changes and not with species or resistance changes. Factors underlying the persistent increase in post-candidemia mortality are unclear.

## Teaser

Post-candidemia mortality has increased since the beginning of COVID-19, alongside changes in *Candida* species and increasing antifungal resistance. Increased mortality in this study was associated with an older, more chronically and acutely ill population contracting candidemia, and not associated with changes in species or resistance patterns.

*Candida* spp. bloodstream infections (BSIs) are one of the most common causes of BSI among hospitalized and critically ill patients and have an associated 30-day mortality of 30%–40% [1–4]. From 2000 to the late 2010s, several large US studies reported that candidemia either declined or remained stable in incidence [4–7]. However, a recent nationwide study described an increased incidence of candidemia since the COVID-19 pandemic [8].

Along with an increase in incidence, there has been a shift in *Candida* spp. distribution*. C. albicans*, frequently the predominant *Candida* species, has declined in incidence over the past 20 years [9–12]. Meanwhile, some studies, particularly in North America and Europe, report that *C. glabrata* [9, 11–13] and *C. parapsilosis* [11, 14] are increasingly common, although these trends vary between continents [15]. Additionally, the potentially multidrug-resistant *C. auris*, which emerged globally in 2009 and in the United States in 2013 [16], markedly increased in incidence during the COVID era [17].

Certain *Candida* species were increasingly resistant to antifungals, particularly azoles, before the pandemic [11]. Recent studies suggest that COVID accelerated this trend, particularly among *C. parapsilosis* [18–22]. Additionally, resistance to the first-line echinocandin antifungals has increased [23], although resistance remains lower than among azoles [24]. Notably, after COVID, there has been an increase in echinocandin resistance in *C. auris*, a species often innately fluconazole resistant [17]. Not all data support the notion that antifungal resistance is increasing, however: a 2025 CDC surveillance study showed no increase in azole or echinocandin resistance in any *Candida* spp. over the last 10 years [4].

Multiple large studies have reported an increase in mortality rates associated with candidemia during COVID [4, 8]. Similar increases in mortality during COVID have been reported in bacteremia patients [25, 26]. This observed increase in mortality coincides with the well-documented shift in *Candida* species and increase in antifungal resistance that may have been accelerated by the pandemic. In a large healthcare system in Houston, TX, with a quaternary center as well as multiple community hospitals, we tracked post-candidemia mortality (defined as death within thirty days of positive *Candida* blood culture) from 2017 to 2025 to determine whether there was a similar increase in mortality during COVID and how those trends changed postpandemic. We also reported changing patterns in species and antifungal resistance to determine if those changes were responsible for any observed increase in mortality. We compared candidemia patients between 3 time periods: 2017–2019 (pre-COVID, Period 1), 2020–2022 (the height of COVID, Period 2), and 2023–2025 (post-COVID height, Period 3). Additionally, we compared mortality after candidemia to mortality after *Staphylococcus aureus* and Enterobacterales bacteremia, to better understand if trends in candidemia mortality are unique or in line with broader developments in BSI outcomes.

## METHODS

### Patient Population

This study was approved by the Houston Methodist Institutional Review Board (IRB # PRO00037862). We included data from patients admitted to 1 of 8 hospitals at a hospital system in Houston, Texas. Our primary analysis focused on patients with a BSI due to *Candida* spp. (or newly designated genera including *Pichia, Clavispora, Candidozyma, Meyerozyma*, and *Kluyveromyces*) between January 1, 2017 and December 31, 2025. We additionally assessed mortality among patients with a BSI due to Enterobacterales (genera *Citrobacter*, *Cronobacter*, *Enterobacter*, *Escherichia*, *Klebsiella*, *Raoultella*, *Morganella*, *Proteus*, *Providencia*, *Salmonella*, and *Serratia*) or *S. aureus* in the same time frame.

### Data Extraction

We extracted demographic (age, sex, race, and ethnicity), clinical variables (intensive care unit [ICU] status, clinical scoring system values, and lab values), and antifungal susceptibility testing results for patients with a blood culture positive for *Candida* spp. ICU-onset candidemia was defined as patients who were in ICU when the positive culture was drawn. All clinical variables were assessed in the 48 hours prior to culture collection. We used the sequential organ failure assessment (SOFA) score (including individual components) at the episode level and Elixhauser Comorbidity Index (ECI) at the patient level to assess acute illness severity and comorbidity burden, respectively, across periods [27–29]. We also analyzed comorbidities of interest including heart failure, peripheral vascular disease, complicated hypertension and diabetes, chronic pulmonary disease, renal failure, liver disease, and obesity.

Antifungal testing was performed at the local clinical microbiology laboratory. Resistance was defined by the Clinical Laboratory and Standards Institute (CLSI) breakpoints [30], or, for *C. auris*, Centers for Disease Control and Prevention (CDC) proposed breakpoints [31]. Species that do not have established antifungal resistance breakpoints were analyzed at the patient and episode level but not included in the antifungal resistance analysis.

In alignment with a recent CDC surveillance study on candidemia [4], for basic demographic information and reported comorbidities, we limited our query to first *Candida* blood culture per patient. For clinical variables and outcomes, we analyzed unique episodes of candidemia. As in the CDC study, any *Candida* positive blood culture within 30 days of the index culture was considered part of same candidemia episode. Polymicrobial *Candida* spp. that grew from the same blood draw were considered 1 episode, but all *Candida* spp. were included in analysis of changes in *Candida* speciation and resistance. Antifungal resistance was analyzed at the level of individual culture susceptibilities without applying the 30-day episode definition, with duplicate same-day cultures growing the same *Candida* spp. counted once. We applied the same “episode” rules to the bacterial cultures that were applied to *Candida*: any culture taken with 30 days was part of the same Enterobacterales or *S. aureus* episode.

### Statistical Analysis

Our data were broken up into periods: 2017–2019 (pre-COVID, Period 1), 2020–2022 (the height of COVID, Period 2), and 2023–2025 (post-COVID height, Period 3). For the analysis, we compared mortality rates for candidemia episodes versus morality rates of *S. aureus* and Enterobacterales bacteremia across periods. We selected these 2 classes as they represent 2 distinct and common BSI types among hospitalized and critically ill patients [32]. We then compared characteristics of patients with candidemia between periods 1, 2, and 3. Initial analysis for overall variation across periods was conducted using Pearson's χ^2^ test for categorical variables, Welch's ANOVA for continuous variables, and Kruskal–Wallis for ordinal variables. Fisher's exact test was used instead of chi square when any cell count was <5. We additionally conducted pairwise testing to analyze differences between periods, using χ^2^ (or Fisher's) for categorical variables, Student's *t*-test for continuous variables, and Mann–Whitney *U* test for ordinal variables. Additionally, we tested for linear trend in mortality across periods using multivariable logistic regression with study period modeled as an ordinal variable. Statistical significance was set at *P* ≤ .05.

We determined risk factors for 30-day mortality among patients with candidemia by univariable logistic regression. We then created a multivariable logistic regression model to determine if mortality increased in periods 2 and 3 after adjusting for baseline demographics, comorbidities, and severity of illness based on preselected, clinically relevant risk factors for mortality among patients with candidemia. All data were analyzed using R version 2025.09.02.

## RESULTS

### Study Population

We identified 1386 unique episodes of candidemia from 1275 patients in our cohort. There were 345 patients in period 1 (2017–2019; 380 episodes), 466 patients in period 2 (2020–2022; 502 episodes), and 464 patients in period 3 (2023–2025; 504 episodes). Across periods, the mean age was 64.5 years (±15.0), 46.4% of patients were female, 61.3% were white, and 19.1% were Hispanic or Latino (Table 1). Mean patient age was the same in periods 1 and 2 (P2 vs P1: 64.1 vs 62.7 years, *P* = .23) but increased in period 3 (P3 vs P2: 66.1 vs 64.1 years, *P* = .04). The proportion of patients who were Hispanic or Latino was higher in period 2 compared with period 1 (P2 vs P1: 22.2% vs 13.2%, *P* = .002) and remained elevated in period 3 (P3 vs P2: 20.0% vs 22.2%, *P* = .44). There was no difference in race or gender between the 3 periods.

**Table 1.** Demographics and Comorbidities of Patients With Candidemia Across 3 Time Periods

	All Patients (N = 1275)	Period 1, 2017–2019 (N = 345)	Period 2, 2020–2022 (N = 466)	Period 3, 2023–2025 (N = 464)	*P* Value*
Age, years, mean (SD)	64.5 (15.0)	62.7 (16.0)	64.1 (14.2)	66.1 (14.8)	.02
Gender, N (%)					1.00
Female	592 (46.4%)	161 (46.7%)	216 (46.4%)	215 (46.3%)	
Male	683 (53.6%)	184 (53.3%)	250 (53.6%)	249 (53.7%)	
Race, N (%)					.93
White	781 (61.3%)	213 (61.7%)	290 (62.2%)	278 (59.9%)	
Black	375 (29.4%)	94 (27.2%)	135 (29.0%)	146 (31.5%)	
Asian	62 (4.9%)	21 (6.1%)	22 (4.7%)	19 (4.1%)	
Other	57 (4.5%)	17 (4.9%)	19 (4.1%)	21 (4.5%)	
Hispanic or Latino	243 (19.1%)	46 (13.3%)	104 (22.3%)	93 (20.0%)	.006
ECI, median (IQR)	9 (5, 13)	8 (5, 12)	9 (5, 13)	11 (6, 15)	<.001
CHF	498 (39.1%)	125 (36.2%)	160 (34.3%)	213 (45.9%)	.002
PVD	386 (30.3%)	73 (21.2%)	128 (27.5%)	185 (39.9%)	<.001
HTN, complicated	596 (46.7%)	146 (42.3%)	195 (41.8%)	255 (55.0%)	<.001
CPD	421 (33.0%)	114 (33.0%)	142 (30.5%)	165 (35.6%)	.44
DM, complicated	535 (42.0%)	116 (33.6%)	194 (41.6%)	225 (48.5%)	<.001
Renal failure	552 (43.3%)	143 (41.4%)	201 (43.1%)	208 (44.8%)	.82
Liver disease	362 (28.4%)	83 (24.1%)	114 (24.5%)	165 (35.6%)	<.001
Obesity	460 (36.1%)	82 (23.8%)	158 (33.9%)	220 (47.4%)	<.001

Abbreviations: CHF, congestive heart failure; CPD, chronic pulmonary disease; DM, diabetes mellitus; ECI, Elixhauser Comorbidity Index; HTN, hypertension; PVD, peripheral vascular disorder.

^*^ *P* value compares all 3 periods.

Median ECI score remained the same from period 1 to period 2 (P2 vs P1: 9 [IQR 5–13] vs 8 [IQR 5–12], *P* = .17) but increased in period 3 (P3 vs P2: 11 [IQR 6–15] vs 9 [IQR 5–13], *P* < .001). The proportion of patients with peripheral vascular disease (P2 vs P1: 27.5% vs 21.2%, *P* = .046; P3 vs P2: 39.9% vs 27.5%, *P* < .001), complicated diabetes (P2 vs P1: 41.6% vs 33.6%, *P* = .01; P3 vs P2: 48.5% vs 41.6%, *P* = .02), and obesity (P2 vs P1: 33.9% vs 23.8%, *P* < .001; P3 vs P2: 47.4% vs 33.9%, *P* < .001) significantly increased stepwise from period 1 to 3. In addition, the proportion of patients with congestive heart failure (P2 vs P1: 34.3% vs 36.2%, *P* = .23; P3 vs P2: 45.9% vs 34.3%, *P* < .001), complicated hypertension (P2 vs P1: 41.8% vs 42.3%, *P* = .99; P3 vs P2: 55.0% vs 41.8%, *P* < .001), and liver disease (P2 vs P1: 24.5% vs 24.1%, *P* = .93; P3 vs P2: 35.6% vs 24.5%, *P* < .001) remained stable from period 1 to period 2 but increased in period 3.

### Candidemia Episode Characteristics

ICU-onset candidemia episodes increased from period 1 to period 2 (P2 vs P1: 40.6% vs 31.3%, *P* = .02) (Table 2). There was no difference in median SOFA score (*P* = .51) or laboratory variables across periods.

**Table 2.** Clinical Variables for Candidemia Episodes Across 3 Time Periods

	All *Candida* Episodes (N = 1386)	Period 1, 2017–2019 (N = 380)	Period 2, 2020–2022 (N = 502)	Period 3, 2023–2025 (N = 504)	*P* Value*
In ICU during culture, N (%)	497 (35.9%)	119 (31.3%)	204 (40.6%)	174 (34.5%)	.03
SOFA score, median (IQR)	5 (3, 8)	5 (3, 8)	6 (3, 8)	5 (3, 8)	.51
SOFA: Central nervous, median (IQR)	1 (0, 2)	1 (0, 2)	1 (0, 1)	1 (0, 2)	.93
SOFA: Cardiovascular, median (IQR)	1 (0, 2)	0 (0, 2)	1 (0, 2)	1 (0, 2)	.14
SOFA: Respiratory, median (IQR)^b^	1 (1, 1)	1 (1, 1)	1 (1, 1)	1 (1, 1)	.23
SOFA: Coagulation, median (IQR)	0 (0, 1)	0 (0, 1)	0 (0, 1)	0 (0, 2)	.97
SOFA: Liver, median (IQR)^c^	0 (0, 1)	0 (0, 1)	0 (0, 1)	0 (0, 1)	.81
SOFA: Renal, median (IQR)	1 (0, 2)	1 (0, 2)	1 (0, 2)	1 (0, 2)	.62
BUN, mg/dL, mean (SD)	44.1 (31.2)	42.0 (32.0)	46.6 (33.9)	43.1 (27.5)	.16
Creatinine, md/dL, mean (SD)	2.08 (1.97)	2.08 (1.96)	2.17 (2.11)	1.99 (1.84)	.51
Lactic acid, mmol/L, mean (SD)^a^	3.87 (4.19)	3.38 (3.55)	4.26 (4.79)	3.83 (3.94)	.09
WBC, k/µL, mean (SD)	14.4 (10.5)	13.9 (10.4)	14.9 (10.6)	14.2 (10.6)	.50
Hgb, g/dL, mean (SD)	9.02 (2.13)	9.01 (2.02)	9.13 (2.19)	8.93 (2.16)	.52
Platelet, k/µL, mean (SD)	199 (140)	198 (146)	197 (135)	200 (141)	.99
30-d mortality, N (%)	385 (27.8%)	75 (19.7%)	160 (31.9%)	150 (29.8%)	<.001

Abbreviations: BUN, blood urea nitrogen; Hgb, hemoglobin; ICU, intensive care unit; SOFA, sequential organ failure assessment; WBC, white blood cells.

^*^ *P* value compares all 3 periods.

^a^Lactic acid values missing for 402 patients: 149 in period 1, 160 in period 2, and 93 in period 3.

^b^Respiratory SOFA sub-component missing for 286 patients: 107 in period 1, 90 in period 2, and 89 in period 3.

^c^Liver SOFA sub-component missing for 212 patients: 82 in period 1, 73 in period 2, and 57 in period 3.

**Figure 1.**
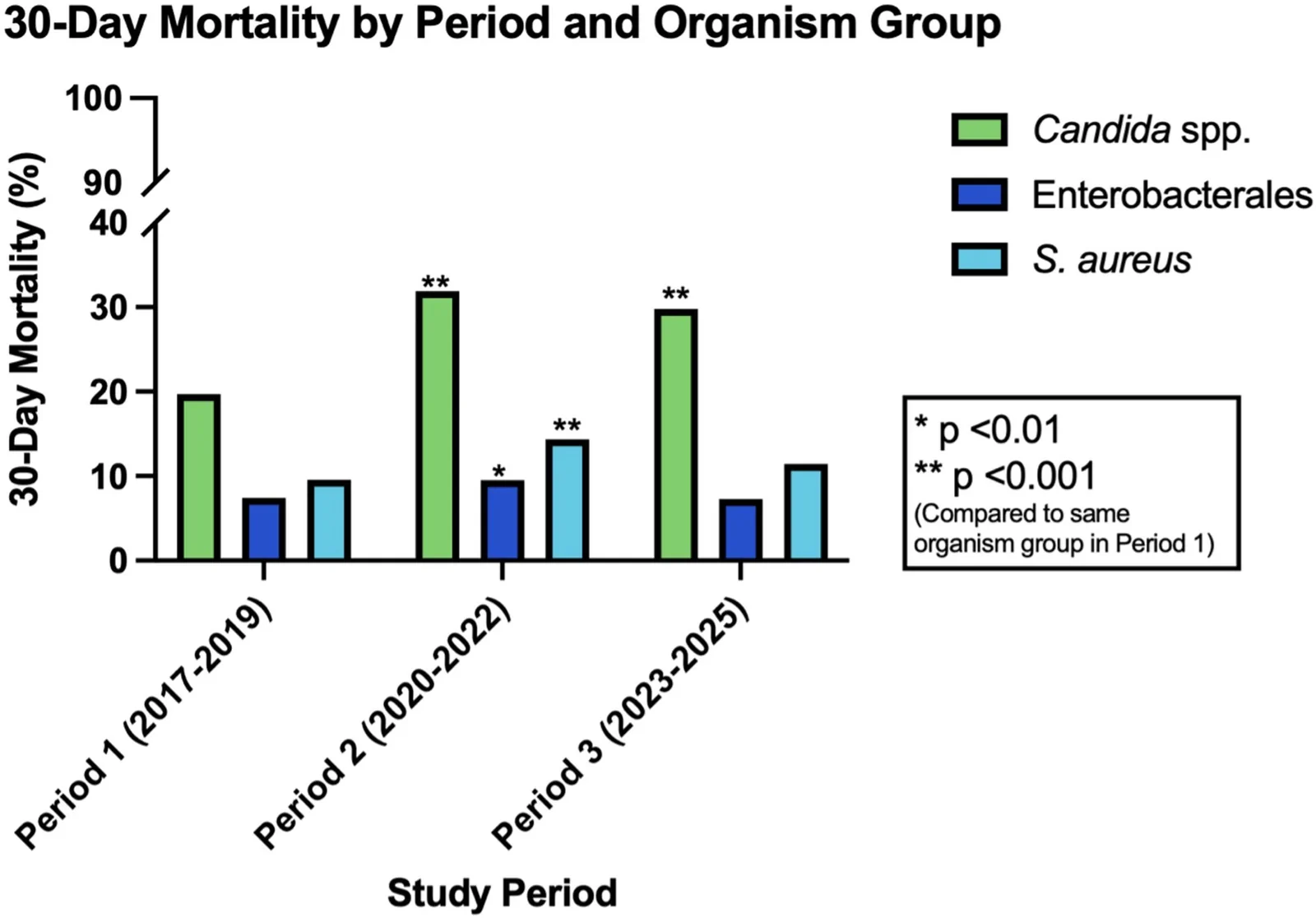
30-day mortality by bloodstream infection type across 3 separate periods, 2017–2025.

Overall 30-day post-candidemia mortality across periods was 27.8%. Among candidemia episodes, 30-day mortality increased significantly from period 1 to period 2 (P2 vs P1: 31.9% vs 19.7%, *P* < .001) and remained elevated in period 3 (P3 vs P1: 29.8% vs 19.7%, *P* < .001) (Figure 1). To compare changes in mortality related to *Candida* BSIs versus bacterial BSIs, we analyzed mortality across periods for Enterobacterales and *S. aureus* bacteremia as well. We captured 18 031 Enterobacterales bacteremia episodes: 4652 in period 1, 5753 in period 2, and 7626 in period 3. For *S. aureus* bacteremia, we captured 6460 episodes: 1920 in period 1, 2232 in period 2, and 2317 in period 3. In contrast to the observed trend in candidemia mortality, for Enterobacterales (E) and *S. aureus* (*SA*) bacteremia episodes, 30-day mortality also increased in period 2 versus period 1 (E, P2 vs P1: 9.5% vs 7.4%, *P* = .004; *SA*: 14.3% vs 9.5%, *P* < .001), but mortality returned to pre-COVID levels in period 3 (E: 7.3% vs 7.4%, P3 vs P1, *P* = .28; *SA*: 11.4% vs 9.5%, P3 vs P1 *P* = .19).

### Species Distribution

From the 1386 candidemia episodes, our cohort had 1431 *Candida* isolates (44 episodes of candidemia were polymicrobial for ≥2 *Candida* spp.) (Table 3). Prevalence of *C. albicans* remained stable from period 1 to period 2 (P2 vs P1: 27.1% vs 31.3%, *P* = .19) but decreased in period 3 (P3 vs P2: 20.3% vs 27.1%, *P* = .01). *C. auris* increased from period 1 to period 2 (P2 vs P1: 3.9% vs 0.5%, *P* = .003) and further increased in period 3 (P3 vs P2: 20.5% vs 3.9%, *P* < .001). *C. parapsilosis* prevalence increased from period 1 to period 2 (P2 vs P1: 20.9% vs 15.2%, *P* = .04) but decreased in period 3 (P3 vs P2: 10.4% vs 20.9%, *P* < .001). *C. glabrata*, *C. tropicalis*, and other *Candida* spp. remained stable across periods.

**Table 3.** *Candida Spp.* Distribution Across 3 Time Periods

	All *Candida* spp. (N = 1431)	Period 1, 2017–2019 (N = 387)	Period 2, 2020–2022 (N = 517)	Period 3, 2023–2025 (N = 527)	*P* Value*
*C. auris*	130 (9.1%)	2 (0.5%)	20 (3.9%)	108 (20.5%)	<.001
*C. albicans*	368 (25.7%)	121 (31.3%)	140 (27.1%)	107 (20.3%)	.002
*C. glabrata*	458 (32.0%)	129 (33.3%)	155 (30.0%)	174 (33.0%)	.67
*C. parapsilosis*	222 (15.5%)	59 (15.2%)	108 (20.9%)	55 (10.4%)	<.001
*C. tropicalis*	125 (8.7%)	38 (9.8%)	41 (7.9%)	46 (8.7%)	.81
Other *Candida* spp.	128 (8.9%)	38 (9.8%)	53 (10.3%)	37 (7.0%)	.24

^*^ *P* value compares all 3 periods.

### Antifungal Resistance

We captured 1596 *Candida* isolates to analyze trends in antifungal resistance (Supplementary Table 1). In *C. albicans* and *C. glabrata* isolates, there was no difference in fluconazole resistance across periods. Fluconazole resistance decreased in *C. tropicalis* isolates from period 1 to 2 (P2 vs P1: 10/42 [23.8%] vs 15/29 [51.7%], *P* = .02). Additionally, *C. parapsilosis* fluconazole resistance decreased from period 2 to period 3 (P3 vs P2: 17/65 [26.2%] vs 62/113 [54.9%], *P* < .001). Virtually all *C. auris* isolates (165/166) tested against fluconazole were resistant.

In *C. auris*, there was no difference in micafungin resistance from period 2 to period 3 (P3 vs P2: 32/145 [22.1%] vs 7/22 [31.8%], *P* = .42). Micafungin resistance decreased in *C. glabrata* from period 1 to 2 (P2 vs P1: 4/125 [3.2%] vs 12/93 [12.9%], *P* = .008) and remained low in period 3. Other species were largely micafungin susceptible across periods. Lastly, there was no difference in amphotericin B resistance in *C. auris* isolates between periods 2 and 3 (P3 vs P2: 96/145 [66.2%] vs 13/22 [59.1%], *P* = .63).

### Risk Factors for Mortality

In univariable analysis, both periods 2 (OR 1.90, 95% CI 1.39–2.62) and 3 (OR 1.72, 95% CI 1.26–2.37) were associated with higher risk of mortality compared with period 1 (Table 4). Compared with other *Candida* spp., *C. auris* was associated with decreased mortality (OR 0.57, 95% CI 0.35–0.89). Neither fluconazole resistance (OR 0.78, 0.56–1.06) nor micafungin resistance (OR 0.62, 95% CI 0.27–1.27) were associated with mortality. Age, ICU status, and increased SOFA subscore were associated with higher risk of mortality (Table 4).

**Table 4.** Odds Ratios for 30-Day Mortality for Candidemia Episodes

Characteristic		N Used	Unadjusted Odds Ratio (95% CI)	*P* Value	Adjusted Odds Ratio (95% CI)	*P* Value
Period	1	1386	Reference		…	
	2	1386	1.90 (1.39–2.62)	<.001	1.60 (1.06–2.45)	.03
	3	1386	1.72 (1.26–2.37)	<.001	1.65 (1.08–2.54)	.02
*C. auris*	…	1386	0.57 (0.35–0.89)	.02	…	
Micafungin resistance	…	1089	0.62 (0.27–1.27)	.22	…	
Voriconazole resistance	…	640	0.89 (0.56–1.39)	.62	…	
Fluconazole resistance	…	1101	0.78 (0.56–1.06)	.12	…	
Age^a^	…	1386	1.02 (1.01–1.03)	<.001	1.03 (1.02–1.04)	<.001
Gender	Male	1386	Reference		…	
	Female	1386	0.87 (0.69–1.10)	.25	1.28 (0.93–1.77)	.13
Race	Caucasian	1386	Reference		…	
	Black	1386	0.85 (0.65–1.10)	.22	0.93 (0.64–1.35)	.70
	Asian	1386	0.96 (0.54–1.63)	.87	0.61 (0.29–1.25)	.19
	Other	1386	0.96 (0.54–1.67)	.90	1.12 (0.51–2.41)	.78
Ethnicity	Non-Hispanic	1386	Reference		…	
	Hispanic	1386	1.17 (0.87–1.57)	.30	0.83 (0.53–1.27)	.39
In ICU during culture	…	1386	6.59 (5.10–8.55)	<.001	2.08 (1.46–2.98)	<.001
SOFA: Central nervous^b^	…	1386	1.48 (1.34–1.62)	<.001	1.15 (1.01–1.32)	.04
SOFA: Cardiovascular^b^	…	1386	2.38 (2.08–2.74)	<.001	1.47 (1.21–1.78)	<.001
SOFA: Respiratory^b^	…	1100	0.64 (0.52–0.78)	<.001	0.83 (0.64–1.05)	.14
SOFA: Coagulation^b^	…	1386	1.79 (1.63–1.98)	<.001	1.28 (1.11–1.48)	<.001
SOFA: Liver^b^	…	1174	1.97 (1.75–2.23)	<.001	1.57 (1.34–1.85)	<.001
SOFA: Renal^b^	…	1386	1.24 (1.13–1.35)	<.001	1.20 (1.06–1.35)	.004
ECI^b^	…	1386	0.99 (0.97–1.01)	.42	0.96 (0.93–0.99)	<.001
CHF	…	1386	1.25 (0.99–1.59)	.06	…	
PVD	…	1386	1.19 (0.92–1.53)	.18	…	
HTN, complicated	…	1386	0.87 (0.68–1.10)	.23	…	
CPD	…	1386	0.95 (0.74–1.22)	.71	…	
DM, complicated	…	1386	0.93 (0.73–1.18)	.57	…	
Renal failure	…	1386	0.95 (0.75–1.20)	.66	…	
Liver disease	…	1386	1.28 (0.99–1.65)	.06	…	
Obesity	…	1386	0.88 (0.69–1.12)	.29	…	
BUN^c^	…	1386	1.02 (1.01–1.02)	<.001	…	
Creatinine^c^	…	1386	1.08 (1.02–1.15)	.006	…	
Lactic acid^c^	…	984	1.23 (1.18–1.29)	<.001	…	
WBC^c^	…	1386	1.05 (1.03–1.06)	<.001	…	
HGB^c^	…	1386	0.86 (0.80–0.91)	<.001	…	
Platelets^c^	…	1386	0.99 (0.99–1.00)	<.001	…	

Abbreviations: ICU, intensive care unit; CRRT, continuous renal replacement therapy; SOFA, sequential organ failure assessment; ECI, Elixhauser Comorbidity Index; CHF, congestive heart failure; PVD, peripheral vascular disorder; HTN, hypertension; CPD, chronic pulmonary disease; DM, diabetes mellitus; BUN, blood urea nitrogen; WBC, white blood cells; Hgb, hemoglobin.

^a^Odds of mortality per year increase in age.

^b^Odds of mortality per point increase in SOFA subscore or ECI.

^c^Odds of mortality per point increase in lab value.

After adjusting for demographic characteristics and illness severity in a multivariable logistic regression model (949 total episodes were included in the regression), periods 2 (OR 1.60, 95% CI 1.06–2.45) and 3 (OR 1.65, 95% CI 1.08–2.54) were associated with higher mortality compared with period 1. In an analysis for linear trend across period, there was a significant overall upward trend in mortality across periods (OR 1.26, 95% CI 1.02–1.55). Additionally, age (OR 1.03, 95% CI 1.02–1.04) and ICU status (OR 2.08, 95% CI 1.46–2.98) were independently associated with mortality.

## DISCUSSION

In a cohort of patients with candidemia at a large healthcare system, 30-day mortality increased from 20% before COVID (2018–2020) to ∼30% during COVID (2020–2022) and remained elevated after the pandemic (29.8% from 2023 to 2025). We also observed a shift in *Candida* speciation, where *C. albicans* declined in incidence and *C. auris* increased significantly across periods. We saw minimal changes in antifungal resistance, and neither *C. auris* species nor resistance was associated with mortality. Results of a multivariable analysis suggest that the observed increases in mortality were in part associated with an aging patient population that is more critically ill.

Our mortality rate of ∼30% in periods 2 and 3 is slightly lower but largely aligned with reported 30-day mortality rates in other studies of candidemia, which range from 32% to 38% [2, 3, 33]. Additionally, other studies have also shown an increase in mortality rates over time: notably, a CDC surveillance study showed an increase from 27% mortality in 2017% to 36% in 2021 [4]. Elevated mortality in 2020–2021 has been assumed to be due to the COVID pandemic. However, our cohort is unique for analyzing postpandemic mortality, and we observed that post-candidemia 30-day mortality remained elevated after the pandemic. In contrast, 30-day mortality associated with 2 common, clinically relevant groups of bacteria (Enterobacterales and *S. aureus*) increased during COVID but decreased to prepandemic levels in period 3. The increase in bacteremia-associated mortality during the pandemic may have been related to complications of COVID and the strain on hospital systems in the early pandemic [25, 26]. While those factors may have affected candidemia mortality rates as well, the persistence of elevated candidemia mortality after COVID suggests that some other, additional process is contributing to mortality in these cases. Although we did not control for differences between candidemia and the bacteremia groups, the diverging mortality trends still highlight an important development in candidemia epidemiology. Recent studies have suggested that the patient population contracting candidemia has gotten older and sicker in the past decade [4, 34]. Our data largely supports this development, with the average age of patients who contracted candidemia increasing from periods 1 to 3, along with increasing comorbidity burden.

In addition to characteristics of candidemia patients and episodes, we also analyzed species trends across periods. *C. albicans* decreased stepwise in incidence across periods, from 33% in period 1 down to 21% by period 3. *C. glabrata*, which was almost equivalent in incidence with *C. albicans* in period 1 (∼33%), remained stable across periods, accounting for 28% of all candidemia cases in period 2% and 32% in period 3. This trend in our cohort aligns with other data that has shown *C. albicans* decreasing in incidence over time [9, 11]. Additionally, the incidence of *C. auris* increased significantly across periods. Only 2 cases were reported in period 1, but by period 3, *C. auris* was responsible for over 20% of all candidemia episodes. Only *C. glabrata* and *C. albicans* were more common than *C. auris* in period 3. This expansion seen in our hospital system echoes a global phenomenon: *C. auris* has quickly spread, and outbreaks are difficult to control due to *C. auris*'s ability to persist on the skin and medical devices [35]. Although it is largely acknowledged that *C. auris* has spread rapidly, the reported range on the proportion of candidemia cases caused by *C. auris* varies widely. Our data differs from recent CDC data, which reported that from 2017 to 2021, <1% of all cases in their surveillance sites were due to *C. auris* [4]. Other surveillance programs also report low, but increasing, proportions of *C. auris*: the SENTRY antifungal surveillance program reported that 1.6% of invasive candidiasis episodes in 2022 were due to *C. auris*, up from <1% in 2018–2021 [36]. Many studies focused specifically on individual hospital systems show similarly high proportions (14%–30%) of *C. auris* candidemia [37], particularly after the pandemic [38], which may reflect publication bias by hospital systems with high *C. auris* prevalence. A recent study with nationwide hospital data showed a low overall prevalence of *C. auris* [13].

Our study sought to determine which factors contributed to the increased candidemia mortality burden since the start of COVID-19. Among species, we found that only *C. tropicalis* was associated with a higher risk of death compared with other *Candida spp*. Additionally, mortality in *C. auris*, which was the only species that uniformly increased across time periods, was lower than other *Candida* spp. Both findings are consistent with recent studies that investigated the mortality risk of various *Candida* spp [2, 39]. Antifungal resistance to fluconazole or micafungin was not associated with increased mortality. Similarly, a 2025 study of *C. parapsilosis* BSI specifically found no relation between fluconazole resistance and mortality [40]. These data suggest that another process, besides the emergence of *C. auris* or high rates of antifungal resistance, is driving the increased mortality since COVID.

After our data showed that species changes and antifungal resistance were not driving the increase in post-candidemia mortality, we created a multivariable analysis to determine if the increase was entirely explained by well-documented risk factors for morality in candidemia patients, such as age, ICU status, and elevated SOFA score [2, 41]. While these factors were associated with increased mortality, after adjusting for these variables, we found that periods 2 and 3 were independently associated with increased risk of death compared with period 1. This suggests that, outside of the variables we controlled for, there was some change between periods that is not captured in our data. Although we capture many variables of interest, there are well-known management-related risk factors for post-candidemia mortality not included in our dataset, such as inappropriate antifungal therapy (or total lack of therapy) and CVC retention [42]. Our dataset does not include information on antifungal therapy or on whether CVC was present at any time during infection. CVC presence—and appropriate removal—is particularly relevant in *C. auris* BSI, as *auris* commonly colonizes the skin [35]. It's possible that increases in these variables may additionally contribute to the increased risk of death we observed in periods 2 and 3.

Our study has several limitations, in addition to the possible unmeasured confounders mentioned above. Our data is from a single hospital system, which limits generalizability. However, our hospital system is made up of a large referral hospital and several community hospitals, which should increase the generalizability of our findings. We also do not have data on which candidemia episodes were COVID-associated. Although COVID-associated candidemia is an important and well-reported phenomenon, our study was focused on tracking other markers of illness over time. Next, comorbidities were based on ICD-10 codes, which may be inaccurately documented in some cases. We did not include data on antifungal therapy and are unable to comment on if or how treatment practices changed across the 3 periods. Clinical decision-making around antifungal therapy initiation for patients with candidemia is under-studied, and further research is needed to better understand practices related to antifungal therapy and whether they have changed over time. Lastly, we reported antifungal susceptibility results as determined by our local microbiology laboratory, which may differ from gold-standard testing.

## CONCLUSION

Post-candidemia mortality increased during the COVID era and has remained elevated since. Notably, bacteremia-associated mortality (specifically Enterobacterales and *S*. *aureus* bacteremia) also increased during the pandemic but subsequently returned to pre-COVID levels. Although increased post-candidemia mortality coincided with an increase of *C. auris* BSIs in our cohort, neither the emergence of *C. auris* nor antifungal resistance contributed to the increased mortality in our cohort. Our data, which is retrospective and descriptive, suggests that the increase was in part driven by the population getting older and sicker across periods. However, we show that, in addition to the well-understood risk factors for post-candidemia death, such as age, ICU status, and organ dysfunction, additional, unmeasured variables contributed to the increase in mortality across periods. Further research must be done to better understand the factors leading to increasing mortality in patients with candidemia.

## Supplementary Material

ofag558_Supplementary_Data

## Supplementary Data

Supplementary materials are available at *Open Forum Infectious Diseases* online. Consisting of data provided by the authors to benefit the reader, the posted materials are not copyedited and are the sole responsibility of the authors, so questions or comments should be addressed to the corresponding author.
