# Supplementary material for: Contemporary Trends in Candidemia: Mortality, Species, and Antifungal Resistance Across Three Periods (2017–2025) in Houston, Texas
Source: Open Forum Infect Dis. 2026 Sep 9;13(9):ofag558. doi: 10.1093/ofid/ofag558 (PMC13586760; doi:10.1093/ofid/ofag558)
Supplement: Supplementary file 1 [file ofag558_supplementary_data.docx]

**Supplemental Table 1: Antifungal resistance of *Candida* spp*.* across three time periods**

|  | Period 1,  2017-2019 | Period 2,  2020-2022 | Period 3,  2023-2025 | P value* |
| --- | --- | --- | --- | --- |
| *C. albicans* | | | | |
| Fluconazole | 14/120 (11.7%) | 13/130 (10.0%) | 5/99 (5.1%) | 0.207 |
| Voriconazole | 12/120 (10.0%) | 11/130 (8.5%) | 4/99 (4.0%) | 0.231 |
| Micafungin | 1/88 (1.1%) | 1/130 (0.8%) | 0/99 (0.0%) | 0.743 |
| Multiple drugs+ | 1/120 (0.8%) | 0/130 (0.0%) | 0/99 (0.0%) | 0.628 |
| *C. auris* | | | | |
| Fluconazole | 1/1 (100.0%) | 22/22 (100.0%) | 142/143 (99.3%) | 1.00 |
| Micafungin | 0/1 (0.0%) | 7/22 (31.8%) | 32/145 (22.1%) | 0.552 |
| Amphotericin B | 0/1 (0.0%) | 13/22 (59.1%) | 96/145 (66.2%) | 0.32 |
| Multiple drugs+ | 0/1 (0.0%) | 7/22 (31.8%) | 30/145 (20.7%) | 0.432 |
| *C. glabrata* | | | | |
| Fluconazole | 24/118 (20.3%) | 22/125 (17.6%) | 21/140 (15.0%) | 0.535 |
| Micafungin | 12/93 (12.9%) | 4/125 (3.2%) | 6/144 (4.2%) | 0.009 |
| Multiple drugs+ | 4/118 (3.4%) | 1/125 (0.8%) | 1/144 (0.7%) | 0.257 |
| *C. parapsilosis* | | | | |
| Fluconazole | 25/59 (42.4%) | 62/113 (54.9%) | 17/65 (26.2%) | <0.001 |
| Voriconazole | 19/60 (31.7%) | 59/113 (52.2%) | 13/65 (20.0%) | <0.001 |
| Micafungin | 0/53 (0.0%) | 1/113 (0.9%) | 0/65 (0.0%) | 1.00 |
| Multiple drugs+ | 0/60 (0.0%) | 1/113 (0.9%) | 0/65 (0.0%) | 1.00 |
| *C. tropicalis* | | | | |
| Fluconazole | 15/29 (51.7%) | 10/42 (23.8%) | 9/40 (22.5%) | 0.023 |
| Voriconazole | 13/30 (43.3%) | 10/43 (23.3%) | 6/41 (14.6%) | 0.025 |
| Micafungin | 0/24 (0.0%) | 0/43 (0.0%) | 2/41 (4.9%) | 0.19 |
| Multiple drugs+ | 0/30 (0.0%) | 0/43 (0.0%) | 0/41 (0.0%) | NA |

* P-value compares all three periods.

+Resistance to echinocandin and any azole (fluconazole or voriconazole), in line with CDC surveillance study [4].
